# Arsenic Enhances the Degradation of Middle-Chain Petroleum Hydrocarbons by *Rhodococcus* sp. 2021 Under Their Combined Pollution

**DOI:** 10.3390/microorganisms12112279

**Published:** 2024-11-10

**Authors:** Hongpeng Shi, Chengyan Gong, Meilin Zheng, Yinghao Zhao, Ying Liu, Luyan Ma, Zhipei Liu

**Affiliations:** 1 State Key Laboratory of Microbial Resources, Institute of Microbiology, Chinese Academy of Sciences, No. 1 West Beichen Road, Chaoyang District, Beijing 100101, China; shihongpeng21@mails.ucas.ac.cn (H.S.); zhengml@im.ac.cn (M.Z.); zhaoyh1937@163.com (Y.Z.); liuying@im.ac.cn (Y.L.); 2 University of Chinese Academy of Sciences, Beijing 101408, China; 3 Institute of Subtropical Agriculture, Chinese Academy of Sciences, Changsha 410125, China

**Keywords:** green and sustainable bioremediation, combined pollution, biodegradation, heavy metals/heavy metal-like elements tolerance, *Rhodococcus* sp. 2021

## Abstract

The efficient and green remediation of petroleum hydrocarbon (PH) contamination has emerged as a viable strategy for environmental management. Here, we investigated the interaction between arsenic and PH degradation by *Rhodococcus* sp. 2021 under their combined pollution. The strain exhibited disparate responses to varying concentrations and valences of arsenic. The elevated concentration of arsenic (>100 mg/L) facilitated the degradation of PHs, and there was a positive correlation between arsenic-promoted degradation of PHs and their carbon-chain length. The degradation of PHs changed with arsenic conditions as follows: trivalent arsenic groups > pentavalent arsenic groups > arsenic-free groups (control). Arsenite and arsenate significantly promoted the gene expression of arsenic metabolism and alkane degrading. But unlike arsenite, arsenate also significantly promoted the gene expression of phosphate metabolism. And arsenite promoted the up-regulation of the expression of genes involved in the process of PHs oxidation and fatty acid oxidation. These results highlight the potential of *Rhodococcus* sp. 2021 in the remediation of combined total petroleum hydrocarbon (TPH) and heavy metal pollution, providing new insights into the green and sustainable bioremediation of combined pollution of organic matters such as PHs and heavy metals/heavy metal-like elements such as arsenic.

## 1. Introduction

Oil is indispensable for the development of industry, and most of the items and medicines we used are closely related to petrochemicals. A study reports that oil consumption is expected to rise to 113.3 million barrels per day by 2030 [1]. However, it is precisely this overdependence on oil that should give rise to the problem of oil-induced pollution. The transport and storage of petroleum raw materials and the irrational treatment of wastewater or sludge during the production process can cause multidimensional integrated pollution of the sea, soil, and atmosphere [2]. Through accidental emissions from the petroleum industry, natural seepage, and biological activity, 800 million tons of petroleum hydrocarbons (PHs) are released annually [3]. Oil pollution can negatively impact marine communities and ecosystems, with contaminated waters causing the mortality of marine organisms due to localized hypoxia and exposure to toxic substances, and ecotoxicity spreading more widely as it travels through the food chain [4,5]. Petroleum is a persistent composite pollutant, and its accidental release into the environment can affect the growth and development of plants and animals, as well as biodiversity and functioning, which in turn affects ecologically sustainable development. Continuous and long-term exposure of humans and animals to TPH contamination can cause cancerous lesions, which can seriously affect the development of biological health [6]. Petroleum-contaminated sites are often accompanied by heavy metals, which are biotoxic and can affect functional organisms’ ability to function in the contaminated environment. Plants and animals can bioconcentrate heavy metals, and human consumption of vegetables and livestock products with excessive levels of heavy metals/heavy metal-like elements can lead to poisoning incidents, which can seriously endanger biosecurity and human health [7].

Currently, the commonly used treatment options for site contaminants are removal and transformation through physical removal, chemical removal, biological treatment, and alternating mixed methods of multiple options [8]. However, there are advantages and disadvantages of each of these options in the in situ remediation process. The physical and chemical treatment options can cause changes in soil properties while risking the introduction of new contaminants, and it is difficult to reuse the treated land for agricultural production [9,10]. The use of hydrogen peroxide in a mixture of programs can affect soil biological activity and inhibit microbial degradation [11]. Most importantly, to date, the biggest bottleneck faced during bioremediation of site contamination is the problem of combined pollution of organic contaminants and heavy metals/heavy metal-like elements [12], due not only to the composite contamination of organic matter and heavy metals severely limiting microbial in situ remediation at contaminated sites [13,14], but also the unknown factor of the interactions among microbes, inorganic pollutants, and organic pollutants.

Then, how to carry out green and sustainable remediation in a composite polluted environment without introducing new pollution sources has become a research focus. Bioremediation has the advantage of being economical and environmentally friendly, making it suitable for sustainable remediation. The biodetoxification of combined pollutants is feasible through the development of efficient bacterial agents. In this study, middle-chain *n*-alkanes (C_11_H_24_, C_13_H_28_, C_14_H_30_, C_15_H_32_, C_16_H_34_, C_17_H_36_, C_18_H_38_) and arsenic (arsenite/arsenate) were used as a model for the combined pollution of PHs and HMs/HM-like elements, and *Rhodococcus* sp. 2021, capable of PH degrading and arsenic resistance, was obtained [15]. Herein, the interactions and molecular mechanisms among As, PHs, and strain 2021 were investigated. The results might provide novel clues and provide insights into the interactions among microbes, inorganic pollutants and organic contaminants, and might also be significant for the green and sustainable bioremediation of PH and HM combined pollution sites.

## 2. Materials and Methods

### 2.1. Chemical Reagents and Bacterial Strain

Totally, 7 middle-chain PHs were used: *n*-undecane (*n*-C_11_, ≥97%), *n*-tridecane (*n*-C_13_, ≥99.0%), *n*-tetradecane (*n*-C_14_, ≥98.0%), *n*-pentadecane (*n*-C_15_, ≥96.0%), *n*-hexadecane (*n*-C_16_, ≥96.0%), *n*-heptadecane (*n*-C_17_, ≥95%), and *n*-octadecane (*n*-C_18_, ≥97.0%). All were purchased from Tianjin Chemical Reagent Research Institute Co., Tianjin, China. Total PHs (TPHs) consist of an equal weight of the above PHs, and served as the sole carbon source for the degradation experiments. Sodium arsenite (As^3+^) (90%, Sigma-Aldrich, Saint Louis, MO, USA) and sodium arsenate (As^5+^) (≥98%, Sigma-Aldrich, Saint Louis, MO, USA) were used as trivalent and pentavalent arsenic compounds for the subsequent tests.

*Rhodococcus* sp. 2021 was used, which was isolated from a contaminated site of an old coking plant [15]. The mineral salt medium (MSM) was configured according to the method of Shi et al. [15] and used for degradation tests. LB medium was used for preculture of the strain. For preparation of solid medium, 1.5% (*w*/*v*) agar was added. All medium were autoclaved at 121 °C for 30 min.

### 2.2. Degradation Tests of Total Petroleum Hydrocarbons (TPHs)

The TPH degradation tests were carried out in 50 mL triangular flasks. Cells of the preculture in LB liquid at 30 °C overnight on a rotation shaker (160 rpm) were harvested by centrifugation at 6000× *g* for 10 min, then washed three times with sterile MSM and resuspended in sterile MSM to OD_600_ about 0.80. Next, 200 µL of the cell suspension was inoculated into flask containing 20 mL MSM with TPHs as the sole carbon source and incubated on a rotating shaker at 30 °C and 160 rpm for 5 days or otherwise stated.

The Box–Behnken design method, that is, the Response Surface Method (RSM), was employed to study and optimize the biodegradation conditions for TPHs, as described by Al-Baldawi et al. [16]. The interactions between the main factors including content of TPHs (500, 2500, and 4500 mg/L), temperature (25, 32.5, and 40 °C), initial pH (5.0, 7.0, and 9.0) and NaCl content (0, 2, and 4%, *w*/*v*) were investigated, as well as the response of strain 2021 to TPH biodegradation and bacterial growth under combined pollution.

To investigate the effects of arsenic valence and concentration on the biodegradation of TPHs sodium arsenite and sodium arsenate were selected as trivalent and pentavalent arsenic sources. Arsenic concentrations were set at 0, 25, 50, 100, and 200 mg/L in triplicate for each treatment group. Initial values for concentration of TPHs, temperature, pH, salinity, and arsenic concentration at non-experimental variables were 2500 mg/L, 30 °C, 7.0, 0%, and 200 mg/L, respectively, or otherwise indicated.

### 2.3. Analysis Methods

At the end of the tests, TPHs in the culture were extracted using *n*-hexane. Specifically, the inner wall of the triangular flask was rinsed with 15 mL of *n*-hexane, and then the mouth of the flask was sealed using an organic solvent-resistant plastic film, and placed into a constant temperature ultrasonic extractor for 30 min, and then placed into a shaker at 30 °C, 160 rpm, for 15 min to collect the organic phase, and the process was repeated three times. The collected organic phase was evaporated in a rotary evaporator at 30 °C until ~2 mL remained, and again, the volume was fixed to 10 mL using *n*-hexane. The samples were filtered using 0.22 μm pore size filter prior to the determination, and the TPH content was determined using a gas chromatograph (Agilent Technologies 7890A, Agilent Technologies, Wellington, DE, USA) equipped with HP-5 column [(5%-phenyl)-methylpolysiloxane non-polar column, 60 m × 250 μm × 0.25 μm, Agilent] [17]. GC conditions: 70 °C for 0 min, then 10 °C/min to 310 °C for 6 min with a run time of 30 min.

The biomass was determined by the dry weighing method [18]. After the extraction of TPHs, 3 mL of *n*-hexane was again added, and the organisms were at the interface of the aqueous and organic phases. The cells were pipetted into 10 mL high-temperature-resistant centrifuge tubes that had been constantly weighted, and the organic phase in the centrifuge tubes was volatilized in a fume cupboard, then pre-dried to an anhydrous state in a thermostatic oven at 85 °C, and then dried at 105 °C for 9 h. The tubes were cooled in desiccator and then weighed.

Arsenic valence transformation caused by strain 2021 was evaluated through a 7-day incubation period in 50 mL flasks containing 20 mL of MSM. The reaction solution was subsequently collected for physicochemical factor analysis. As outlined by Shi et al. [15], the flasks were sealed with plastic film and then subjected to ultrasonic disruption for 30 min, which was repeated four times. The resulting solid particles were filtered using filter paper and then filtered using 0.22 µm pore size filter membrane. Subsequently, the filtrate was collected and the pentavalent arsenic was determined using an ion chromatograph (ICS-2100, Thermo Fisher Scientific Inc., Waltham, MA, USA), while the total arsenic was analyzed using ICP-OES (Avio 200, PerkinElmer Inc., Waltham, MA, USA). The trivalent arsenic content was calculated as the difference between the total arsenic and the pentavalent arsenic content.

### 2.4. Transcriptome Analysis

In order to investigate the mechanism of arsenic enhancing the degradation of petroleum hydrocarbons by strain 2021, the experiments were set up with a control group (CK, arsenic-free), and test groups containing 200 mg/L As^3+^ (TAs3) and As^5+^ (TAs5) in triplicate. The concentration of TPHs was 2500 mg/L. The experiments were conducted for 5 days and the bacterial cells were harvested for transcriptome sequencing analysis. Total bacterial RNA was extracted using the TrizoL method and genomic DNA was removed, and the qualified RNA samples obtained were sent to Majorbio Bio-Pharm Technology Co., Ltd. (Shanghai, China). RNA library construction was performed using Illumina^®^ Stranded mRNAPrep, Ligation from Illumina (San Diego, CA, USA), followed by RNA-seq bipartite sequencing using IlluminaNovaSeq6000. Data generated by the Illumina platform were analyzed using Majorbio Cloud Platform (cloud.majorbio.com). The prokaryotic strand-specific transcriptome sequencing raw data have been uploaded to the NCBI database, and the BioProject accession number is PRJNA1123476.

### 2.5. Statistical Analyses

A response surface design and analysis was performed using Design-Expert 12. GraphPad Prism 9.3.1 was used for plotting and a one-way ANOVA statistical analysis of the data.

## 3. Results and Discussion

### 3.1. Influences of Physico-Chemical Factors on the Degradation of TPHs and the Growth of Strain 2021

**Figure 1:**
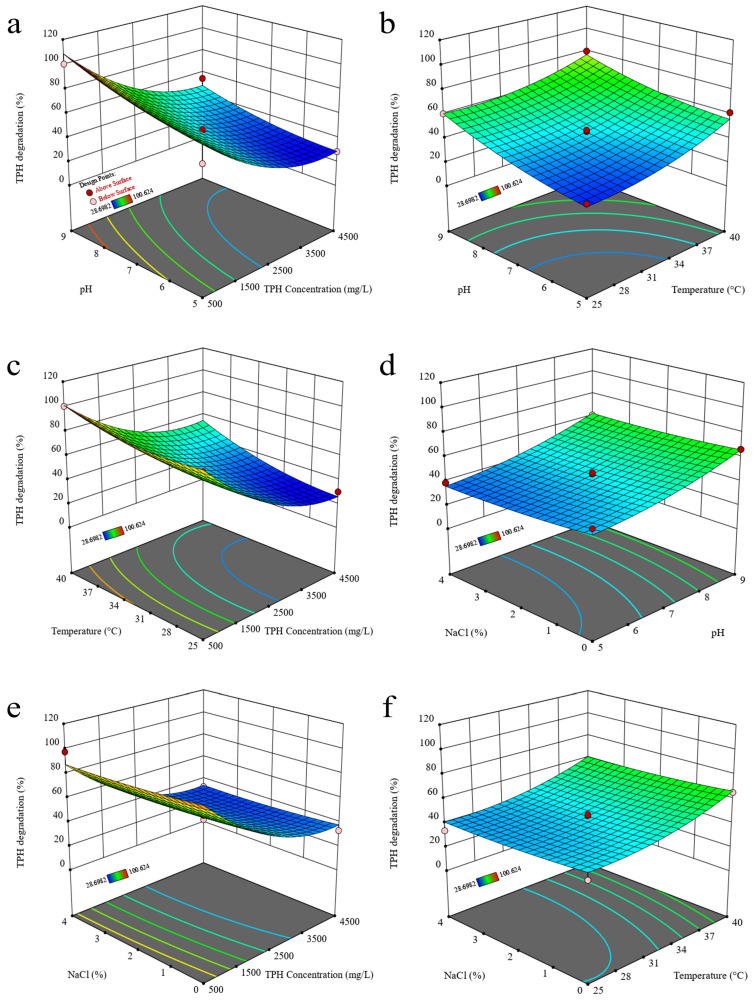
Effects of physico-chemical factors on the degradation of TPHs by strain 2021. Note: Four factors examined in the experiments were TPH concentration, temperature, pH, and NaCl content. The response values were the degradation percentages of TPHs, and the incubation time was 5 days. (**a**) pH and TPH content; (**b**) pH and temperature; (**c**) temperature and TPH content; (**d**) NaCl content and pH; (**e**) NaCl content and TPH content; and (**f**) NaCl content and temperature. Initial values for TPH concentration, temperature, pH, and salinity in non-experimental groups were 2500 mg/L, 32.5 °C, 7.0, and 2%, respectively. The red dots are for design points above the predicted value; the pink dots are for design points below the predicted value.

**Figure 2:**
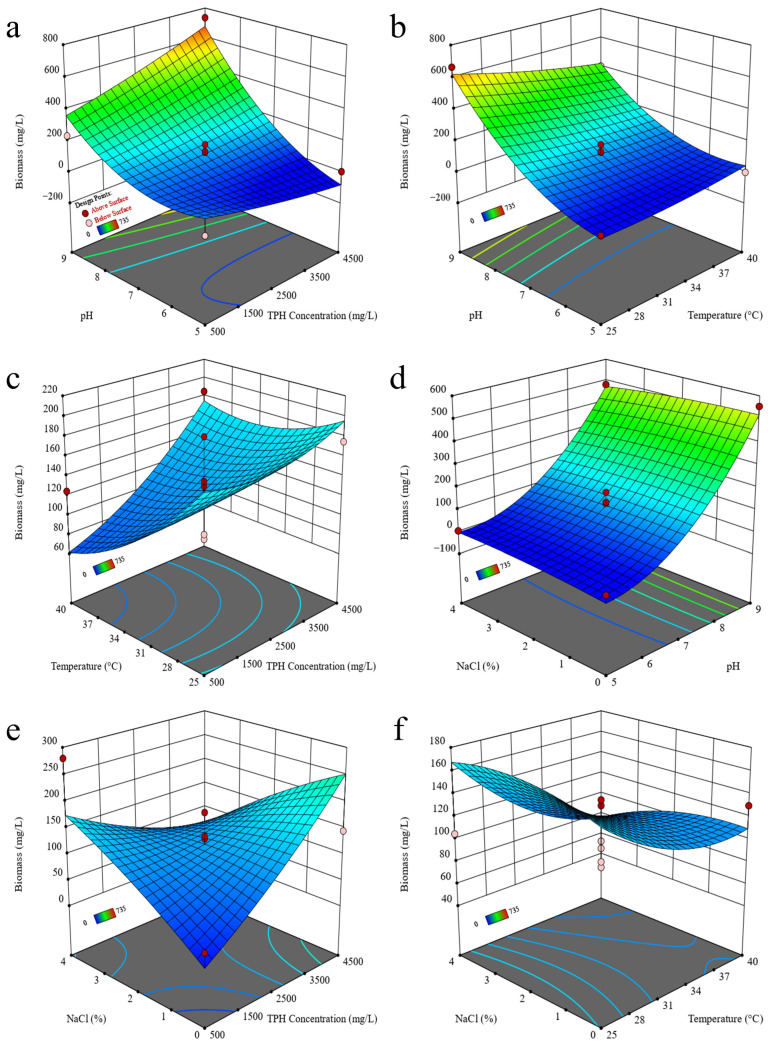
Effects of physico-chemical factors on the growth of strain 2021. Note: Four factors examined in the experiments were TPH concentration, temperature, pH, and NaCl content. The response values were the biomass of strain 2021, and the incubation time was 5 days. (**a**) pH and TPH content; (**b**) pH and temperature; (**c**) temperature and TPH content; (**d**) NaCl content and pH; (**e**) NaCl content and TPH content; and (**f**) NaCl content and temperature.

The degradation of TPHs and the growth (biomass) of strain 2021 were expressed as response values with the changes in TPH concentration, temperature, pH, and NaCl content in two-factor ways. The results showed that the degradation of TPHs was found to increase with the increase in pH, from 38.81–46.79% at pH 5 to 60.86–~100% at pH 9 (Figure 1a–d). The biomass of strain 2021 increased significantly with the increase in pH, from 5 mg/L at pH 5 to 480–555 mg/L at pH 9 (Figure 2d). The degradation of TPHs by strain 2021 exhibited a notable decline with the increase in TPH content, from 97.71–100.07% (500 mg/L) to 32.81–33.26% (4500 mg/L) (Figure 1e). In contrast, the biomass increased from 25 mg/L (500 mg/L of TPHs) to 145 mg/L (4500 mg/L of TPHs) when cultivated in the absence of NaCl (0% NaCl), and decreased from 280 mg/L (500 mg/L of TPHs) to 35 mg/L (4500 mg/L of TPHs) when 4% NaCl was present (Figure 2e). Briefly, those results demonstrated that the concentration of TPHs, temperature, and pH were the primary factors influencing the degradation of TPHs by strain 2021 (Figure 1). The highest degradation of TPHs (100%) was observed at pH 9 and 500 mg/L TPHs (Figure 1a). The impact on the growth (biomass) of strain 2021 was evidenced by a pH of 9 and 4500 mg/L TPHs, which resulted in the highest biomass (735 mg/L) of strain 2021 (Figure 2a).

The biodegradation of TPHs can be enhanced by introducing exogenous bacteria into polluted environments, and bacteria have different removal efficiencies of TPHs in microenvironments with varying levels of pollution [19]. It was found that the biodegradation of TPHs decreased with increasing concentration of TPHs, but the removal efficiency increased with increasing concentration of TPHs. In the organic matter degradation test, bacteria obtained carbon sources for growth between the organic and aqueous phases, and as the concentration of TPHs increased, the area and probability of bacterial contact with the carbon source increased, leading to an increase in bacterial biomass in the microenvironment, and a concomitant increase in the consumption of TPHs for the purpose of pollutant removal. Temperature and pH can cause changes in the activities of enzymes involved in TPH transport and degradation by affecting bacterial motility, bioactivity, and enzyme activity, which in turn cause changes in the efficiency of TPH degradation [13]. Lower temperatures lead to weakened bacterial motility and reduced enzyme activity. Increased temperature results in enhanced bacterial activity and increased enzyme activity, while the solubility of the contaminant increases with temperature, raising the probability of exposure and utilization of the contaminant by organisms [20]. As bacterial growth occurs in large quantities, microenvironmental pH decreases, and changes in microenvironmental acidity and alkalinity affect bacterial morphology, motility, and enzyme activity [13]. Strain 2021 was found to increase its biomass and TPH biodegradation with an increasing pH. TPH degradation increased with increasing temperature, but biomass decreased with increasing temperature in alkaline environments, and temperature had no significant effect on the biomass increase in acidic environments. This is due to the increase in temperature, which leads to enhanced volatility of TPH, lower concentration of TPH, and reduced probability and opportunity for bacterial contact with TPH, resulting in low biomass [21]. Salinity can alter the rate of the biodegradation of TPH and organic matter by affecting microbial cell membranes and enzyme bioactivity, which ultimately affects the geochemical cycling of hydrocarbons [22]. Studies have shown that salinity reduces microbial community size, but actinomycetes were significantly enriched in high-salt environments [22]. In this study, salinity had a weak effect on the biodegradation, but the biomass of strain 2021 varied with salinity when temperature and TPH concentration were variables with salinity, respectively.

### 3.2. Effects of Arsenic Concentration and Valence on the Degradation of TPHs

**Figure 3:**
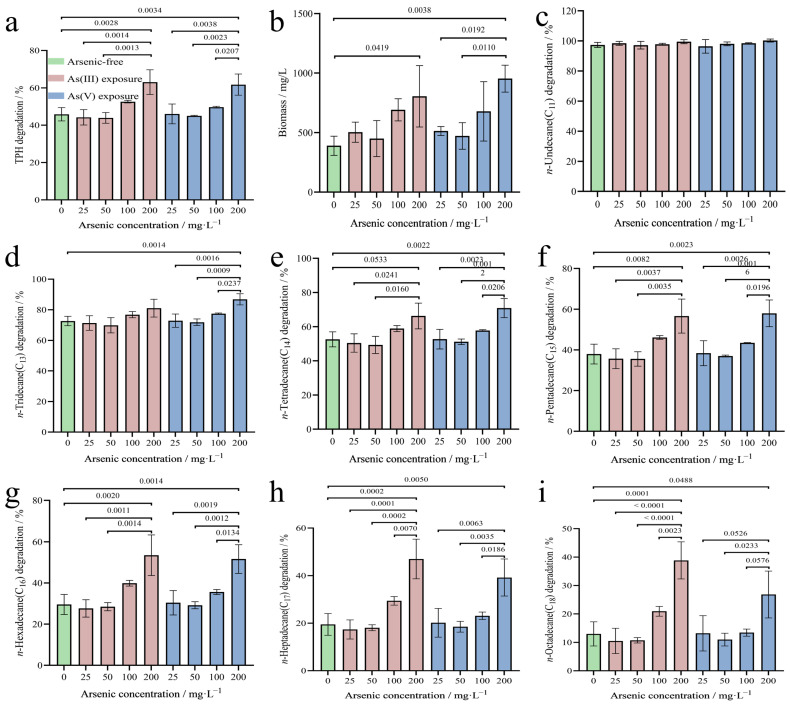
Effect of arsenic valence and content on TPH degradation and biomass of strain 2021. Note: For all groups, experiments were conducted in 50 mL flasks containing 20 mL MSM in triplicate and incubated on a rotating shaker at 30 °C and 160 rpm for 5 days. Initial content of TPHs, temperature, and pH were 2500 mg/L, 30 °C, and 7.0, respectively. (**a**) TPH degradation; (**b**) strain 2021 biomass; (**c**–**i**) degradation percentages of individual *n*-alkanes that comprise the TPHs. The actual *p*-value is indicated on the n-zigzag lines of the bar chart.

The effects of arsenic concentration and valence on the degradation of TPHs and the growth of strain 2021 were investigated under the combined pollution of TPHs and arsenic. The results indicated that the degrading ability and growth of strain 2021 on TPHs were not significantly enhanced by arsenic at low concentrations (i.e., <50 mg/L). Interestingly, high arsenic content (i.e., ≥100 mg/L) could greatly promote the degradation of TPHs by strain 2021 (Figure 3a,b). The degradation of TPHs increased from 43.91 ± 2.35% (50 mg/L As^3+^) and 44.99 ± 0.27% (50 mg/L As^5+^) to 63.07 ± 5.38% (200 mg/L As^3+^) and 61.74 ± 4.64% (200 mg/L As^5+^) (Figure 3a). The degradation of *n*-alkanes in the 200 mg/L trivalent arsenic-exposed group was increased by 2.10 ± 0.69% (*n*-C_11_), 8.43 ± 7.10% (*n*-C_13_), 13.69 ± 9.50% (*n*-C_14_), 18.65 ± 10.48% (*n*-C_15_), 23.92 ± 11.62% (*n*-C_16_), 27.51 ± 10.28% (*n*-C_17_), and 25.86 ± 8.67% (*n*-C_18_), respectively, as compared to the control (arsenic-free) group. In addition, the degradation in the 200 mg/L pentavalent arsenic-exposed group increased by 2.93 ± 1.96% (*n*-C_11_), 14.20 ± 1.65% (*n*-C_13_), 18.28 ± 2.74% (*n*-C_14_), 19.99 ± 3.81% (*n*-C_15_), 22.07 ± 4.66% (*n*-C_16_), 19.71 ± 5.80% (*n*-C_17_), and 13.85 ± 6.48% (*n*-C_18_) (Figure 3c–i). It is of particular importance to note that the degradation-promoting effect of arsenic on strain 2021 was found to be significantly enhanced with the prolongation of the *n*-alkane carbon chain. Furthermore, our results suggested that the higher the concentrations of As^3+^ tested, the greater the degradation-promoting advantage (Figure 3). To date, the interesting phenomenon that arsenic could enhance the petroleum hydrocarbon-degrading ability of a bacterial strain has not been described.

Petroleum extraction smelting and processing sites are subject to multiple environmental organic and HM composite pollutant contamination due to accidental spills and non-standard discharges and stockpiles, which can limit the bioremediation process and efficiency of organic contaminants [23]. Arsenic, as a heavy metal-like element, is potentially biotoxic, and functional microorganisms respond differently to different valences of arsenic. During soil bioremediation, heavy metal stress induces changes in the growth, metabolism, and morphology of microbial communities, leading to a decrease in soil microbial diversity [24]. Interestingly, strain 2021 could efficiently degrade TPHs under the combined pollution of TPHs and arsenic. Furthermore, it is noted that arsenic could significantly promote the degradation of TPHs, with trivalent arsenic promoting slightly better than pentavalent arsenic. Studies on the effects of HMs on microbial metabolism have shown that metabolic pathways such as lipid metabolism, aromatic metabolism and transporter proteins/pumps are enhanced with increasing HMs concentrations [23]. This study corroborates the possibility that arsenic can promote the degradation of TPHs by strain 2021 at the metabolic level. Arsenate [As(V)] is a phosphate analog in cellular processes and interferes with biological processes such as cellular oxidative phosphorylation and ATP production. Arsenite [As(III)] derives its toxicity mainly from its tendency to readily bind to sulfhydryl groups, interfering with the biological functions of sulfur-containing proteins [25]. It has been shown that the bacterial *ars* operon encodes arsenite efflux system, and that resistance to arsenate requires the gene *arsC* to encode arsenate reductase, which reduces pentavalent arsenic to trivalent arsenic, and then detoxifies arsenicals from the cytosol by using arsenite efflux system [26]. ArsR senses and responds to low concentrations of the inducer, which in turn initiates the transcription of *arsB* and *arsC*, and the ArsAB complex reduces intracellular arsenic concentrations to lower levels than if ArsB acted alone, reflecting the higher arsenic resistance of the ATP-driven efflux pump [26]. This also explains at the molecular level that the degradation function of strain 2021 shows inhibition in low arsenic concentration environments, while high arsenic concentration promotes TPH degradation. Most importantly, strain 2021 showed superior degradation activity for longer-chain TPHs at high arsenic concentrations.

### 3.3. Progress Course of Arsenic Enhancing the Degrading Ability and Growth of Strain 2021 Under the Combined Pollution of TPHs and Arsenic

**Figure 4:**
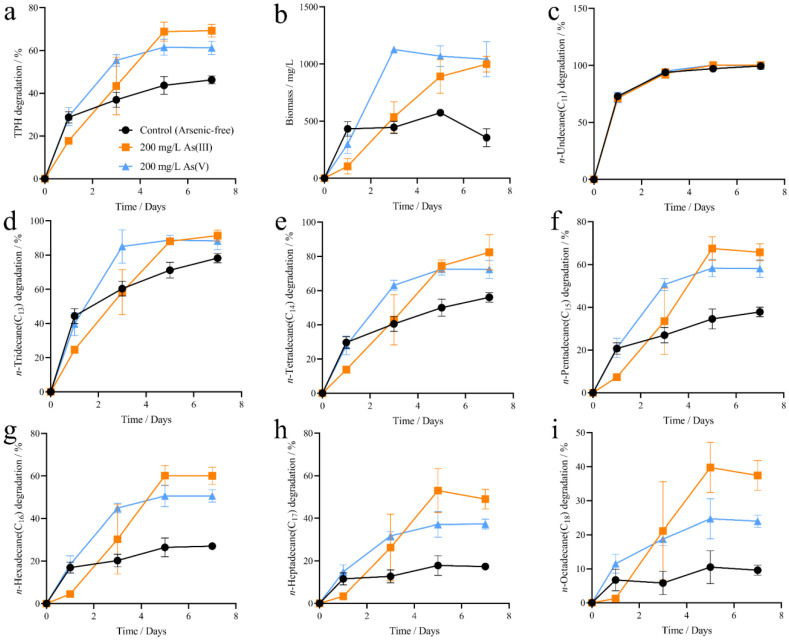
Degradation of TPHs by strain 2021 at different arsenic valence states. Note: TPHs as the sole carbon source. Initial TPH concentration, temperature, and pH were 2500 mg/L, 30 °C, and 7.0, respectively. (**a**) TPH degradation; (**b**) strain 2021 biomass; (**c**–**i**) degradation of individual *n*-alkanes that comprise the TPHs.

Arsenic enhancing the degrading ability and growth of strain 2021 on TPHs was investigated for a week under the combined pollution of TPHs and arsenic, and the culture samples were taken on days 1, 3, 5, and 7 for the analyses of TPH degradation, bacterial growth, and valence change in arsenic. The results showed that on day 1, the degradation of TPHs was 28.76 ± 2.16% (As-free group), 29.10 ± 3.46% (As^5+^ group), and 17.74 ± 0.68% (As^3+^ group), respectively (Figure 4a). The growth (biomass) of strain 2021 was 431.67 ± 52.01 mg/L (As-free group), 298.33 ± 67.37 mg/L (As^5+^ group), and 105.00 ± 54.92 mg/L (As^3+^ group), respectively (Figure 4b). This result suggested that As^5+^ exhibited an inhibition efficiency on the growth of the strain, and As^3+^ an even higher inhibition efficiency on both the degrading ability and growth of strain 2021, at the early stage of the cultivation. On day 3, the degradation of TPHs was 36.92 ± 2.81% (As-free group), 55.37 ± 2.22% (As^5+^ group), and 43.37 ± 10.96% (As^3+^ group) (Figure 4a), and the biomass of the strain reached 445.00 ± 43.20 mg/L (As-free group), 1126.67 ± 20.95 mg/L (As^5+^ group), and 535.00 ± 109.77 mg/L (As^3+^ group) (Figure 4b), respectively. On day 3, in contrast to day 1, both arsenic ions, especially As^5+^, exhibited quite efficient enhancement for both the degrading ability and growth of the strain. On day 7, the degradation of TPHs was 46.25 ± 1.49% (arsenic-free group), 61.20 ± 2.57% (As^5+^ group), and 69.29 ± 2.43% (As^3+^ group), and the biomass of the strain was 355.00 ± 63.77 mg/L (As-free group), 1041.67 ± 125.19 mg/L (As^5+^ group), and 998.33 ± 56.32 mg/L (As^3+^ group), respectively (Figure 4a,b). The results suggested that the enhancing ability of arsenic continued up to the end of the experiments. Regarding individual *n*-alkanes, the results indicated that on day 7, the degradation of the substrates in As^3+^ groups increased by 13.14 ± 4.76% (*n*-C_13_, Figure 4d), 26.36 ± 9.55% (*n*-C_14_, Figure 4e), 27.91 ± 5.05% (*n*-C_15_, Figure 4f), 32.94 ± 4.73% (*n*-C_16_, Figure 4g), 31.65 ± 4.86% (*n*-C_17_, Figure 4h), and 27.78 ± 4.49% (*n*-C_18_, Figure 4i), as compared to the control groups (As-free). In As^5+^ groups, the degradation increased by 10.01 ± 4.15% (*n*-C_13_, Figure 4d), 16.32 ± 4.43% (*n*-C_14_, Figure 4e), 20.28 ± 3.40% (*n*-C_15_, Figure 4f), 23.51 ± 2.06% (*n*-C_16_, Figure 4g), 19.95 ± 1.27% (*n*-C_17_, Figure 4h), and 14.34 ± 0.83% (*n*-C_18_, Figure 4i). It is noted that As^3+^ was a bit more efficient than As^5+^ in promoting the degrading ability and growth of strain 2021 under the combined pollution of arsenic and petroleum hydrocarbons. Interestingly, the results further indicate that the degradation-promoting effects of arsenic favors the longer carbon chain petroleum hydrocarbons, that is, the longer the carbon chain of the middle-chain petroleum hydrocarbons, the stronger the degradation-promoting effects of arsenic (Figure 4).

The presence of arsenic or the valence of arsenic affects the degradation of TPHs by strain 2021. The biodegradation of TPH was higher in the pentavalent arsenic-treated and control groups than in the trivalent arsenic-treated group during the pre-test period, which was attributed to the fact that trivalent arsenic has lower adsorption and higher bioavailability and mobility than pentavalent arsenic, resulting in a stronger trivalent arsenic biotoxicity than pentavalent arsenic [27]. With the activation of arsenic-tolerant gene expression in the strain, the presence of trivalent arsenic continued to stimulate the sustained expression of *ars* gene cluster, conferring higher arsenic tolerance of the strain. The presence of arsenic induces the overproduction of intracellular reactive oxygen species (ROS), leading to an increase in intracellular redox potential. The microbial degradation of TPHs requires the insertion of -OH to activate the hydrocarbon oxidation process, in which firstly the terminal methyl group of TPHs is oxidized to alcohols, which are further oxidized to aldehydes and finally to fatty acids. The generated fatty acids are further processed through β-oxidation to form acetyl CoA, which finally enters the lipid metabolic pathway for catabolism [11,28]. The addition of trivalent arsenic promoted the oxidation process of alkanes, so the final degradation rate of TPHs by strain 2021 would be higher than that of the pentavalent arsenic-treated group and the control group. The longer the carbon chain of TPHs, the higher the energy required for the alkane oxidation process, and so the oxidative toxicity effect caused by the bioavailability of trivalent arsenic was subsequently weakened, which in turn led to the higher bioavailability of TPHs by strain 2021.

### 3.4. Transformation of the Valence of Arsenic

**Figure 5:**
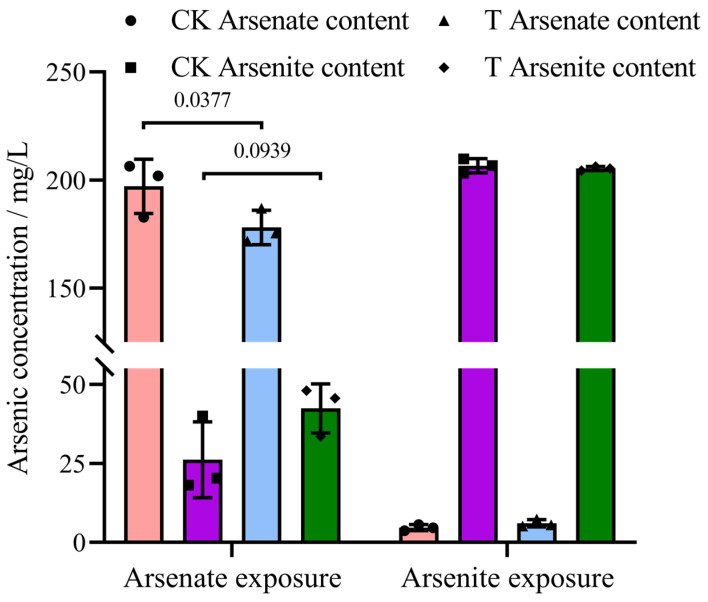
Transformation of arsenic valence in different groups. Note: CK, abiotic control group; T, inoculated group. Sodium arsenate dibasic heptahydrate and sodium arsenite were used, respectively. The initial content of TPHs, pH, and temperature were 2500 mg/L, 7.0, and 30 °C. The red, purple, blue and green columns represent the abiotic control pentavalent arsenic content, the abiotic control trivalent arsenic content, the inoculated group pentavalent arsenic content and the inoculated group trivalent arsenic content, respectively. Data were analyzed using two-way ANOVA statistical method. The actual *p*-value is indicated on the n-zigzag lines of the bar chart.

As revealed above, arsenic could promote the degrading ability of middle-chain PHs of strain 2021. Therefore, how did arsenic valence change during arsenic-promoted degradation of TPHs with different valence arsenic groups under the combined pollution of TPHs and arsenic? In this case, the transformation of the valence of arsenic in the experiments mentioned above was investigated. The results showed that the As^5+^ content (178.13 ± 6.47 mg/L) of the experimental groups was much lower than that (197.10 ± 10.26 mg/L) of the As^5+^ control group (*p* < 0.05). Meanwhile, the As^3+^ production (42.41 ± 6.31 mg/L) of the experimental groups was higher than that (26.16 ± 9.81 mg/L) of the As^3+^ control groups (*p* = 0.0939). However, in the As^3+^ experiments, there was no significant difference in the As^3+^ or As^5+^ levels between the experimental groups and the abiotic control group (*p* > 0.05) (Figure 5). Furthermore, these results strongly suggested that As^5+^ could be reduced to As^3+^ by strain 2021, but As^3+^ was hardly oxidized to As^5+^ by the strain, under our experimental conditions. The mechanism for this phenomenon needs to be studied in the future.

The valence state of arsenic in microenvironments is subject to a number of factors, including the type of carbon source present, the concentration of arsenic, pH levels, temperature, the redox state, and the presence of electron donors [13,29,30]. During the course of biological evolution, microorganisms have evolved a range of natural defense mechanisms against arsenic, including the processes of oxidation/reduction, chelation, and toxicity sequestration [31,32]. During the degradation of TPHs, strain 2021 could be tolerant to different valence forms of arsenic, which was attributed to the activation of intracellular arsenic antagonist gene (*arsRABC*) and transporter protein-encoding gene expression in response to high arsenic exposure. The strain is capable of undergoing intracellular arsenic redox and chelation reactions to resist the cytotoxic effects of arsenic, and is also able to excrete toxicants out of the cell through exocytosis.

### 3.5. Transcriptome Analysis of Strain 2021 Under the Combined Pollution of TPHs and Arsenic

**Figure 6:**
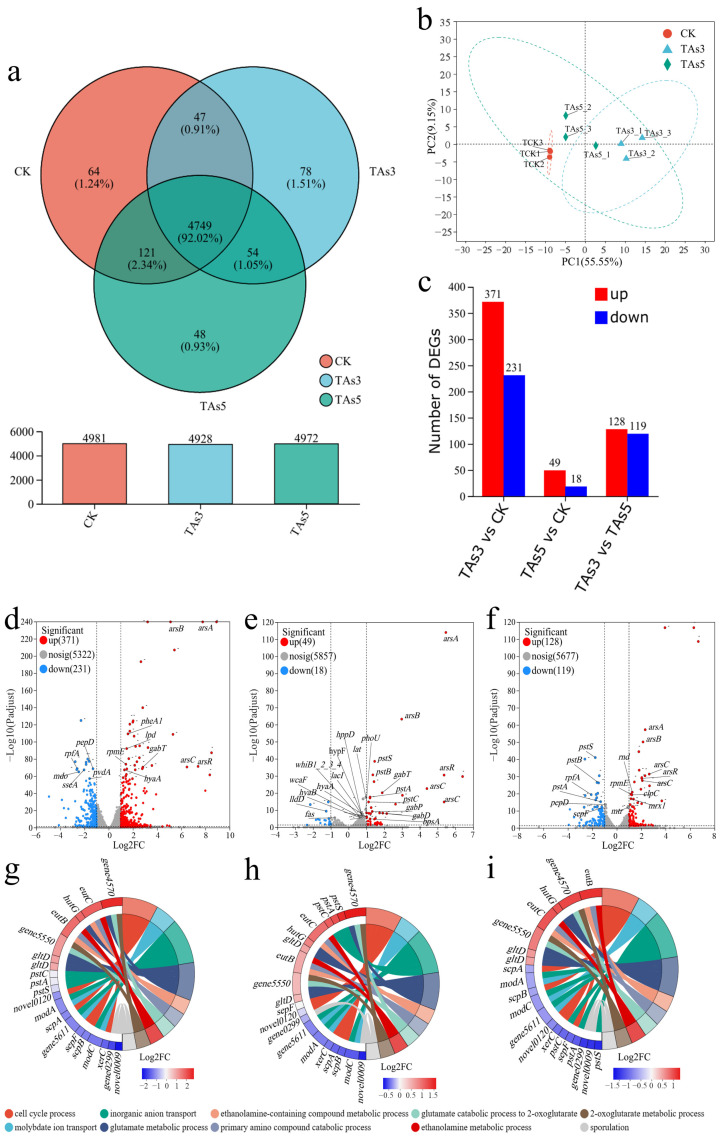
Effect of arsenic valence on transcriptome expression of strain 2021. Note: CK, free-arsenic groups; TAs3, As^3+^ (200 mg/L) groups; TAs5, As^5+^ (200 mg/L), and all other test conditions were consistent. (**a**) Venn analysis and gene expression analysis between groups; (**b**) principal component analysis (PCA) based on the gene expression of each group; (**c**) differential analysis of gene expression between groups, with the red color representing the up-regulation, the blue color representing the down-regulation, horizontal coordinates representing differential groupings, and vertical coordinates representing the number of differential genes; (**d**–**f**) the volcano plot analyses of the differences in gene expression between TAs3 and CK, TAs5 and CK, and TAs3 and TAs5 groups, respectively, with the red points representing genes significantly up-regulated, the blue points representing genes significantly down-regulated, and the gray points representing genes that are non-significantly different; the horizontal coordinate is the logarithm of the fold change value (FC value) of the gene expression between the two groups, the vertical coordinate is the negative logarithm of the statistical test value (*p* value) of the difference in gene expression, and the higher the *p* value is, the more significant the difference in expression is; (**g**–**i**) the GO terms corresponding to the top 10 most significantly enriched GO terms for the differentially expressed genes between the groups of TAs3 and CK, TAs5 and CK, and TAs3 and TAs5, respectively. The left side of the circle shows the gene names. log2FC > 0: gene expression is up-regulated, and the larger the value, the larger the gene differential expression fold. Log2FC < 0: gene expression is down-regulated, and the smaller the value, the larger the gene differential expression fold. A log2FC value closer to 0 indicates that the gene’s differential expression fold is smaller. The right side of the circle shows GO terms information for significant enrichment of differential genes.

In order to reveal the molecular mechanism of the degradation-promoting efficiency of arsenic for strain 2021, the transcriptomes of the strain grown under different conditions including As-free (CK), As^3+^ (TAs3), and As^5+^ (TAs5) were analyzed. The results (Figure 6) indicated that there were 4981, 4928, and 4972 genes transcribed and annotated in agreement with the genome of strain 2021, in CK, TAs3, and TAs5 groups, respectively. Among them, 4749 genes were shared-transcript in all three groups, accounting for 92.02% of the total genes defined in its genome (Figure 6a). The number of specifically transcribed genes in the CK, TAs3, and TAs5 groups was 64, 78, and 48, accounting for 1.24%, 1.51%, and 0.93% of the total genes, respectively (Figure 6a). The results of a principal component analysis (PCA) showed that the CK and TAs3 groups were better differentiated on PC1 (55.55% explained variance) at a confidence interval of 95%, whereas the TAs5 group was similar to both the CK and TAs3 groups (Figure 6b). The number of genes up-regulated or down-regulated in the TAs3 group in comparison with the CK group was 371 and 231, respectively. The number of genes up-regulated or down-regulated in the TAs5 group in comparison with the CK group was 49 and 18, respectively. The number of genes up-regulated or down-regulated in the TAs3 group in comparison with the TAs5 group was 128 and 119, respectively (Figure 6c). A differential analysis of gene expression between the groups showed that arsenic affected the gene expression and regulation in strain 2021, with As^3+^ having a significantly higher intervention effect than As^5+^ (Figure 6b,c).

In detail, the transcription of the genes involved in arsenic, amino acid, and energy metabolism, including *arsR*, *arsA*, *arsB*, *arsC*, *gabT*, *pheA1*, *lpd*, *hyaA*, and *rpmE*, were up-regulated, especially those involved in arsenic metabolism, such as *arsR* was up-regulated 90.05-fold, *arsA* (+123.70-fold), *arsB* (+19.43-fold), *arsC* (+138.43-fold), and *gabT* (+3.97-fold); however, the genes related to protein metabolism were significantly down-regulated, such as *rpfA* was down-regulated 10.89-fold, *sseA* (−7.30), *pepD* (−4.37), and *pvdA* (−5.35) in the TAs3 group in comparison with the CK group (positive numbers in parentheses represent the fold up-regulated and negative numbers represent the fold down-regulated) (Figure 6d). The transcription of the genes *arsR* (+33.78), *arsA* (+36.17), *arsB* (+6.49), *arsC* (+33.79), *pstS* (+2.30), *pstB* (2.15), *gabT* (3.06), *gabP* (2.11), *gabD* (2.74), and *bpsA* (1.86) was significantly up-regulated; but the gene *lldD* (−2.90), a lipid transport related gene, was significantly down-regulated in the TAs5 group as compared to the CK group (Figure 6e). As^3+^ significantly increased the transcription of the genes related to arsenic and energy metabolism, RNA transcription and processing, and oxidoreductase activity, such as *arsR* (+2.67), *arsA* (+3.42), *arsB* (+2.99), *arsC* (+4.10), *clpC* (1.59), *rnd* (1.61), and *mrx1* (1.50), and significantly decreased the transcription of the genes related to phosphate transport, protein hydrolysis, and cell division processes, including *pstS* (−5.05), *pstA* (−3.74), *pstB* (−4.24), *rpfA* (−4.37), *pepD* (−2.82), and *sepF* (−3.54) in the TAs3 group as compared to the TAs5 group (Figure 6f). The GO (Gene Ontology) functional significance enrichment analysis of the genes revealed that exposure to trivalent arsenic resulted in the down-regulation of the expression of the genes *pstS*, *pstA*, *pstC*, *modA*, *modC*, and *gene5611* involved in inorganic anion transport in strain 2021 compared to the CK group, whereas it promoted the up-regulation of expression of the genes *gltD*, *hutG*, *gene4570*, and *gene5550* involved in glutamate metabolism and α-ketoglutarate metabolism, and the up-regulation of the expression of the genes *eutB* and *eutC*, which promote ethanolamine metabolism (Figure 6g). The exposure to pentavalent arsenic resulted in the up-regulation of the expression of genes *pstS*, *pstA*, and *pstC* and the down-regulation of the expression of genes *gene5611*, *modA*, *modC*, those genes involved in the transport of inorganic anions (Figure 6h). In comparison with the exposure to pentavalent arsenic groups, trivalent arsenic promoted the up-regulation of the expression of genes for glutamate, α-ketoglutarate, and ethanolamine metabolism, but suppressed the expression of genes involved in inorganic anion transport (Figure 6i).

**Figure 7:**
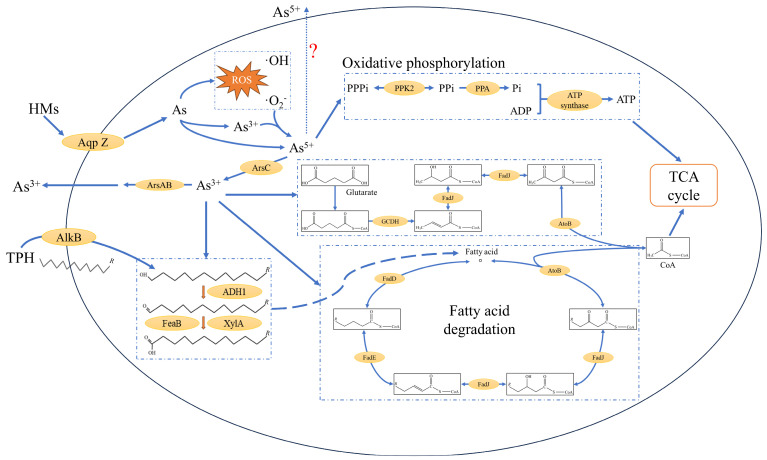
Metabolism of degradation of TPHs by strain 2021 under the combined pollution of TPHs and arsenic. Note: alkane oxidation, fatty acid degradation, and oxidative phosphorylation metabolic processes refer to the KEGG pathway “www.genome.jp/kegg/pathway.html (25 July 2024)”. The question mark(?) represents the mechanism of exocytosis of pentavalent arsenic in *Rhodococcus* sp. 2021 remains to be elucidated.

The exposure of strain 2021 to the combined pollution of PHs and arsenic triggers an adaptive response to meet the energy source and cellular homeostasis required for strain growth (Figure 7). PHs are hydroxylated by alkane 1-monooxygenase (AlkB), and after the introduction of hydroxyl groups to alkanes, they are oxidized by NAD(P)-dependent alcohol dehydrogenase (ADH1) to form aldehydes, which are further oxidized by aldehyde dehydrogenase family protein (FeaB and XylA) to form carboxyl groups. After the oxidation of PHs to fatty acids, they undergo a β-oxidation tandem reaction to remove acetyl coenzyme A step by step, and the final acetyl coenzyme A generated enters the tricarboxylic acid (TCA) cycle process. The presence of As^3+^ promoted the expression of the genes *adh1* (+3.38), *feaB* (+1.05), and *xylA* (+4.84) encoding for ADH1, FeaB, and XylA during fatty acid generation. The expression of *fadD* (+5.14), *fadE* (+2.77), *fadJ* (+1.52), and *atoB* (+4.25), genes encoding long-chain fatty acid-CoA ligase, acyl-CoA dehydrogenase family protein, 3-hydroxyacyl-CoA dehydrogenase family protein, and acetyl-CoA C-acyltransferase involved in the β-oxidation reaction, was also promoted by As^3+^. Notably, the presence of As^5+^ promoted the expression of *pstS*, *pstA*, *pstB*, and *pstC*, genes encoding the phosphate transporter protein PstS, PstA, PstB, and PstC, whereas the presence of As^3+^ inhibits the expression of genes encoding proteins involved in phosphate transport and oxidative phosphorylation. ATP produced during oxidative phosphorylation can enter the TCA cycle for cellular energy metabolism together with acetyl coenzyme A produced during fatty acid β-oxidation. The exposure of strain 2021 to arsenic-containing environments induces intracellular oxidative stress, generating large amounts of reactive oxygen radicals, while cells activate the expression of the gene *oxyR* encoding the LysR family transcriptional regulator in order to maintain homeostasis. LysR family proteins contain a pair of cysteine residues that can be oxidized by hydrogen peroxide to form disulfide bonds, which in turn activate the expression of the downstream alkyl hydroperoxide reductase encoding gene *ahpF*. Trivalent arsenic can promote the expression of strain 2021 gene *oxyR* (+1.28), and also affect the activity of LysR family proteins by acting on the cysteine sulfhydryl site, which in turn affects the intracellular redox state.

Bacteria can convert PHs into fatty acids, which in turn undergo catabolic and anabolic processes via the fatty acid metabolic pathway. The catabolism of fatty acids can serve as a source of cellular energy, and anabolism is associated with intracellular amino acid production [33]. The presence of arsenic promotes the degradation of PHs, which is related to the biological role of arsenic. The presence of arsenic and its valence affects the expression of genes encoding functional proteins in strain 2021. Arsenate, as a phosphate analog, can be involved in the regulation of phosphate metabolism and energy metabolism during cellular biological processes [34]. Arsenite has a propensity to chelate with sulfhydryl groups [35], which can affect the biometabolic processes of sulfur-containing amino acids such as cysteine and glutamate, which are involved in the biosynthesis of functional proteins such as antioxidants, and consequently lead to variability in the expression of the encoded genes [36]. The presence of arsenite greatly enhances differential gene expression compared to differential gene expression in arsenic-free microenvironments, whereas arsenate has relatively little effect on strain 2021 differential gene expression. Arsenite affected the expression of protein-coding genes for arsenic detoxification metabolism, protein translation, glutamate metabolism, redox processes, and aromatic compound synthesis processes in cells, whereas arsenate mainly affected the expression of protein-coding genes for the processes of phosphate metabolism, arsenic detoxification metabolism, and glutamate metabolism in strain 2021. Compared with the arsenate treatment group, the strain differential genes mainly focused on the expression of protein-coding genes for arsenic detoxification metabolism, protein metabolism process, cellular redox process, and protein-coding genes involved in the regulation of precursor tRNA modification and protein translation. Compared with the other treatment groups, the differences in cellular metabolism of strain 2021 in the arsenite treatment group were mainly focused on the processes of glutamate metabolism, α-ketoglutarate metabolism, and ethanolamine metabolism. Glutamate produces α-ketoglutarate through the action of glutamate dehydrogenase, and α-ketoglutarate is a key intermediate product linking glucose metabolism and amino acid metabolism. The lower the abundance of ethanolamine, a metabolite that reports the redox state of the cell, the more oxidative the intracellular environment is and the easier it is for the oxidative catabolism of aliphatic hydrocarbons to be metabolized [34]. Ethanolamine metabolism can provide carbon and nitrogen sources for cells and participate in phospholipid synthesis, and its metabolic process is closely linked with phospholipid metabolism, which together form the basis of cell membrane synthesis [37,38].

## 4. Conclusions

As global industrialization accelerates, the dependence of humanity on petroleum and its chemical products is becoming increasingly serious. However, the problem of petroleum hydrocarbon pollution is also becoming increasingly prominent. Consequently, the efficient and environmentally friendly treatment of the combined pollution caused by petroleum hydrocarbons released into water or terrestrial environments due to accidental or improper storage has become a significant concern. In this study, *Rhodococcus* sp. 2021 was demonstrated to have high salt and arsenic tolerance, and exhibited the capacity to function as a PH degrader in high-salt and high-arsenic-concentration environments. Furthermore, the presence of arsenic could enhance the middle-chain PH-degrading ability of *Rhodococcus* sp. 2021, and this phenomenon was mainly due to the up-regulation of genes involved in arsenic metabolism and PH-degradation. These results provide new insights into the interactions among microbes, organic pollutants, and inorganic pollutants, and also the green and sustainable bioremediation of the combination of petroleum hydrocarbon pollution and heavy metals/heavy metal-like element pollution caused by oil spills.

## Data Availability

The original contributions presented in the study are included in the article, further inquiries can be directed to the corresponding authors.
